# A Cross-National Reliability Study of Catatonia in Individuals with Neurodevelopmental Disorders

**DOI:** 10.1007/s10803-025-06817-9

**Published:** 2025-04-16

**Authors:** Mohammad Ghaziuddin, Neera Ghaziuddin, Carina Gillberg, Elisabeth Fernell, Valdemar Landgren, Christopher Gillberg

**Affiliations:** 1 Department of Psychiatry, University of Michigan, Ann Arbor, MI 48109-0277 USA; 2 Gillberg Neuropsychiatry Center, University of Gothenburg, Gothenburg, Sweden

**Keywords:** Catatonia, Neurodevelopmental disorders, Autism spectrum disorder (ASD), Cross-national, Reliability

## Abstract

Catatonia is a neuropsychiatric disorder characterized by abnormalities of movement, communication, and behavior, often accompanied by a disturbance of mood, thought and perception. If untreated, it may lead to serious complications, including death. Although often described in adults with schizophrenia and severe mood disorders, it can also occur in persons with neurodevelopmental disorders (NDD), particularly autism spectrum disorder (ASD). Since its diagnosis is more likely to be missed by clinicians with limited experience, we attempted to determine if experienced psychiatrists can make a reliable diagnosis of catatonia in a group of patients with NDD. Twenty patients with catatonia with ASD/NDD (12 males 8 females; age range 13–34 years; mean 17.5; SD 4.8), diagnosed by two American psychiatrists, were re-evaluated by a group of Swedish psychiatrists. In the initial round, agreement was reached in 15 (75%) cases. However, a careful review of additional material and several virtual discussions were required to reach agreement in the remaining 5 (25%) of cases. The diagnosis of catatonia in individuals with NDD is challenging, even for experienced clinicians. Reasons for the lack of diagnostic agreement in the five patients are discussed to highlight the barriers for accurately diagnosing catatonia in patients with NDD.

## Introduction

Catatonia is a serious psychiatric disorder characterized by a disturbance of movement, communication and behavior. First described by Karl Kahlbaum in 1874 over a century ago (Fink, 2011), it has often been conceptualized as a subtype of schizophrenia. However, studies in the last two decades have established that it can occur in several conditions, including in infections, exposure to toxins, metabolic diseases, autoimmune and neoplastic disorders, and after intense psychological trauma. When the cause is not clear, a diagnosis of catatonia not otherwise specified is now allowed (APA, DSM-5, 2013). However, despite increasing awareness, catatonia is often missed in clinical practice. Thus, Ghaziuddin and colleagues (2012) found that among one hundred randomly selected adolescents admitted to a psychiatric unit, out of the 18 patients (17.8%) who displayed 3 or more symptoms according to the DSM-5 criteria checklist for catatonia, only two (11%) were correctly diagnosed. Instead, subjects were likely to be diagnosed with intermittent explosive disorder, unspecified psychosis, aggressive behavior, or intellectual disability (ID). Similarly, in a group of 87 patients with autism spectrum disorder (ASD), aged 12–25 years, using a newly developed measure, the Attenuated Behavior Questionnaire completed by a caregiver, Breen and Hare (2017) found that 42 (48.3%) met the criteria for catatonia, although only half had been previously clinically diagnosed with it.

The problem of missing the diagnosis of catatonia is also common in non-psychiatric settings. Llesuy and colleagues (2018) conducted a retrospective survey of adult medical inpatients using DSM-5 criteria for catatonia and identified 133 cases. Out of these, 79 (59%) had not been previously clinically diagnosed with catatonia despite the presence of 3 or more DSM-5 catatonia symptoms. Similar findings have been shown in emergency room settings. Thus, Anand and colleagues in a neurology emergency service found that none of the 12 patients (100%) with a discharge diagnosis of catatonia had received that diagnosis on or before admission (Anand et al., 2019).

Among patients with neurodevelopmental disorders (NDD), most of the research has been done in individuals with ASD. Based on the findings of three studies, Wing and Shah (2006) concluded that as many as 17% of individuals with ASD, over the age of 15 years attending a diagnostic center in the UK met the criteria for catatonia (Wing & Shah, 2006). Despite this, the comorbidity of catatonia with ASD continues to be missed by psychiatrists and other specialists (Ghaziuddin et al., 2012; Breen & Hare, 2017; Llesuy et al., 2018; Anand et al., 2019). This could be due to several reasons. First, most clinicians do not routinely screen for catatonia in NDD, partly due to a lack of awareness of the comorbidity and partly due to diagnostic challenges. Second, remarkable similarities exist between the features of catatonia and those of ASD/NDD. Thus, many patients with ASD at baseline may show abnormal movements such as posturing and stereotypies; communication problems such as echolalia and spontaneous utterances; and behavioral problems such as aggression; all of which can also occur in catatonia. Third, since standardized rating scales for catatonia are infrequently used, the core features of catatonia may be overlooked.

In addition to the similarities in the symptoms of catatonia and NDD, other factors can also complicate the diagnosis. For example, an exclusively psychological conceptualization of symptoms, such as negativism and gegenhalten (involuntary proportional resistance to passive movement of the extremities), may be mistaken for uncooperative behavior and result in a missed diagnosis of catatonia (Oldham, 2019). Similarly, impulsivity and aggression may be wrongly attributed to operant conditioning (reinforcement by parents and other caregivers) or to other reasons (Ghaziuddin et al., 2012). Underdiagnosis of catatonia may lead to suboptimal or incorrect treatment which can affect the long-term outcome of the patient (Breen & Hare, 2017; Llesuy et al., 2018). Untreated catatonia can result in severe complications, including malignant catatonia which is associated with a mortality rate of about 10% (Tuerlings et al., 2010). Differentiating between catatonia and ASD/NDD is also important because the main intervention for ASD is behavior therapy and social skills training, while that of catatonia is pharmacotherapy and/or pharmacotherapy with electroconvulsive therapy (ECT). Because lack of experience and training is often assumed to be the primary reason for missing the diagnosis of catatonia in ASD/NDD, we hypothesized that experienced psychiatrists can reliably diagnose catatonia in this group of patients. To test this hypothesis, we compared the reliability of diagnosis between two teams of experienced psychiatrists, one based in the USA and the other in Sweden.

## Methods

### Sample

The sample consisted of a group of patients with neurodevelopmental disorders (NDD) referred to an academic psychiatry center in the USA. For the purpose of this study, NDD was defined as a group of conditions characterized by an impairment in social or physical development, learning, language, or behavior, starting very early in life. Common examples include ASD, intellectual disability (ID), cerebral palsy, and many other syndromes with ID, such as Down syndrome and Fragile X syndrome.

Patients aged at least 13 years, of both genders, who met the above definition were eligible to participate in the study. The lower age limit of 13 years was chosen because the risk of catatonia increases after puberty in individuals with NDD (Wing & Shah 2006). From this group with NDD, individuals with a history of recent worsening of behavioral symptoms or emergence of new symptoms of severe behavioral deterioration with accompanying motor abnormalities and communication problems were identified by a checklist of symptoms and clinically examined. Patients who could not participate in the study due to absence of reliable informants were excluded, as were those with severe aggression towards self or others; or with other safety concerns.

Experienced clinicians were defined as those who had had at least 5 years of clinical experience in the diagnosis of NDD and catatonia and had also published on the topic in peer-reviewed journals. Since most of the recent studies on catatonia have been published from North America and Europe, two teams of clinicians were chosen, one based in the USA and the other in Europe (Sweden) with each team having similar experience in NDD and catatonia.

### Clinical Assessment

A detailed history of development and of past and present psychiatric history was obtained in all cases from caregivers. Relevant biological tests and school and social agency records were also examined. Details of past pharmacological and psychological treatments were obtained from parents/caregivers and from previous care providers if the parent/caregiver was unable to provide this information. Referral letters from past providers, when available, were also reviewed. Examination of each case included a comprehensive psychiatric history with emphasis on a *recent worsening of baseline symptoms or emergence of novel* symptoms observed by a parent/caregiver, based on the patient’s baseline. A physical examination was completed including a check for symptoms of catatonia commonly described in the literature. Screening was done for less commonly reported motor symptoms, such as tics (Dhossche et al., 2010), tremors, dystonia (Ishizuka et al., 2022), and changes in handwriting (Partl et al., 2011). Items from the Bush Francis catatonia rating scale (BFCRS) were used to supplement the history, including a change in the level of activity, gait, eye-blink rate, and emergence of abnormal motor phenomena such as waxy flexibility (resistance to being moved), rigidity (increased muscle tone), and gegenhalten (involuntary proportional resistance to movement).

### Measures

#### DSM-5 Criteria

The DSM-5 lists 12 symptoms of catatonia including catalepsy, waxy flexibility, stupor, agitation, mutism, negativism, posturing, mannerisms, stereotypies, grimacing, echopraxia and echolalia (APA, 2013). All of these were checked in the cases included here.

#### BFCRS

This is a widely used instrument for catatonia although it is not specifically designed for patients with NDD. It consists of 23 items. Presence or absence of symptoms is assessed using items 1–14 while the severity is determined by the total score of 23 items (Bush et al., 1996).

#### Reliability Protocol

In the pilot phase, six case vignettes, prepared by two US psychiatrists, were sent to the Swedish colleagues who were asked if the patients had catatonia. Three patients had catatonia and three did not. All the vignettes were correctly diagnosed by the four Swedish psychiatrists and consensus was reached between the two teams. Subsequently, de-identified data in about 1000 words was abstracted from the electronic records on 20 subjects and shared with the Swedish team who remained blind to the diagnosis. Consensus agreement was achieved amongst the four Swedish investigators who then sent their results to another investigator (LT) who was not part of the clinical team, to review the protocol, examine the data, and confirm the diagnosis. The results were mailed back to the US team. The two teams then met on Zoom on two occasions to discuss the cases, resolve differences, and reach a consensus diagnosis. E-mail contact was maintained throughout the study period.

#### IRB Approval

Approval from the IRB board in the US, where the individuals were receiving clinical care, was obtained for conducting the study and for sharing de-identified data.

### Data Analytic Plan

Percentage agreement across the two teams of investigators was calculated.

## Results

Two American psychiatrists, each with a minimum of 15 years’ experience in the diagnosis and treatment of NDD and catatonia, diagnosed twenty patients with DSM-5 catatonia (APA, 2013) supplemented by the Bush Francis Catatonia Rating Scale (BFCRS, Bush et al., 1996). Fifteen out of the 20 (75%) cases were “correctly” identified by the Swedish group in the initial round. One of the four Swedish psychiatrists correctly identified all the cases. However, contrary to the hypothesis, full agreement between the teams was not reached initially in five cases (25%). To examine the factors contributing to the disagreement, these five cases are summarized in the table 1 below:

**Table 1:** Patients (25%) in whom initial diagnostic agreement of catatonia was not reached

No	Sex, Age	ID^1^	ASD^2^	Notes	BFCRS^3^ score	Lorazepam trial: highest dose/ response	Comorbid Diagnoses	Family History
#6	M, 15yrs	No	Yes	Incomplete	10	Lorazepam given. Dose and response unclear. ECT^4^ given	Depression and Psychosis	Bipolar Disorder and suspected suicide in a close relative
#9	F, 12yrs	Yes	Yes	Details about workup not given	Not recorded	Likely poor response. ECT given	Anxiety Disorder, Impulse control disorder, SIB^5^	Family history of anxiety disorder
#14	M, 13yrs	Yes	Yes	Admission note diagnosis: Mood Disorder	15	Dose unknown. Poor response. ECT given	Mood Disorder. Past diagnosis of Psychosis.	Family history of mood and anxiety disorders
#16	M, 16yrs	Yes	Yes	Incomplete	25	11 mg daily. Poor response ECT not given	OCD^6^. Pt non-verbal	Family psychiatric history denied
#17	M, 34yrs	Yes	ASD traits	Full details not given	20	4 mg daily/ mild response. ECT not given	ASD and Down syndrome	Family psychiatric history not recorded

ID^1^ = Intellectual Disability; ASD^2^ = Autism Spectrum Disorder; BFCRS^3^ = Bush Francis Catatonia Rating Scale

ECT^4^ = Electroconvulsive Therapy; SIB^5^ = Self Injurious Behavior; OCD^6^ = Obsessive Compulsive Disorder

## Discussion

The main finding of the study is that two teams of experienced psychiatrists did not agree with each other about the diagnosis of catatonia in five (25%) of the cases. Complete agreement in all the 20 cases was eventually reached between the two teams, but only after extensive discussions and examination of additional data. This underscores problems with the reliability of the diagnosis of catatonia in young persons with NDD, faced even by experienced clinicians.

Further analysis of the five cases in whom initial diagnostic agreement was not reached revealed interesting findings. First, we identified that to achieve a diagnostic consensus a *detailed description of symptoms* under the areas of motor, speech and behavior disturbance was essential as was documenting why other diagnoses had been excluded. The limit of 1000 words selected to describe the case in the initial round, could have been insufficient to describe all the symptoms. Second, we found that documenting a *change* in symptom-severity from baseline behavior to the presenting symptoms of catatonia was difficult yet essential for achieving diagnostic consensus. Lack of a clear history of change in symptom frequency and/or severity, could have led to the clinical features being attributed to the underlying NDD rather than to catatonia, by a process of *“diagnostic overshadowing*,*”* in which clinicians attribute new symptoms of a disorder to a pre-existing condition (Hallyburton, 2022; Reinfield and Gill 2023). For example, in one patient behavioral problems were attributed to the existing Down syndrome and ASD, rather than to superimposed catatonia. Third, presence of comorbid conditions could have complicated the diagnosis of catatonia. Indeed, the greater the number of comorbid conditions, the greater the likelihood of catatonia being missed. Thus, four of the five patients in whom consensus was initially not reached, had ID and other co-existing conditions that may have interfered with the diagnosis of catatonia by the Swedish team. In addition to ID, other conditions that could have influenced the diagnostic decision-making were a strong history of psychological trauma, mood and disruptive behavior disorders, and of a strong family history of major mental illness. Finally, all patients where consensus was not reached required ECT and had more prominent symptoms of agitation, restlessness or overactivity, than patients who presented with predominantly psychomotor retardation. This symptom presentation with agitation and restlessness, which is sometimes referred to as excited catatonia (Rasmussen et al., 2016), underscores additional barriers to the diagnosis of catatonia in agitated and overactive patients (Llesuy et al., 2018).

Challenges unique to the diagnosis of catatonia in ASD/NDD, unlike that associated with adult psychiatric disorders, such as depression, include practical difficulties in obtaining a detailed history because patients often have severe communication and behavioral deficits. In these patients, therefore, obtaining information from parents and collateral sources is critical to establish a *change* of behavior from baseline. Another potential barrier to an accurate diagnosis of catatonia is its well-recognized waxing and waning nature of symptoms, which may occur even within an hour (Walther & Stirk 2016) and which may be attributed to temper tantrums or outbursts in individuals with NDD. This further underscores the importance of a systematic assessment which may need to be repeated on more than one occasion. In general, clinicians are inclined to diagnose catatonia when symptoms include those described in historical texts (Fink, 2011; Northoff, 2002). More nuanced presentations of the disorder, as in excited catatonia with frenzied motor activity and aggression towards self or others, may be mistaken for a non-specific behavior disturbance. In such cases sharing of video and images may be necessary to clarify the diagnosis.

Limitations of the study include its small sample size. However, the patients included here represent those who were available at the time of the study. In addition, structured or semi-structured diagnostic interviews could not be used because of the severity of symptoms and the practical difficulties of conducting such assessments in a clinical setting. Video clips of live interviews with patients were not used because of restrictions on data sharing in international studies. Studies using larger samples are required to examine which factors other than training/experience might influence the diagnosis of catatonia in persons with autism/NDD.

The main strength of the study lies in the fact that it is based on a unique group of patients who are at an increased risk of developing catatonia. The reliability of diagnosis was examined between two groups of experts located in different countries. Data shared during the first round was rated blindly, thereby diminishing the confounding group-effects on consensus ratings. Furthermore, clinical assessment which included detailed documentation of symptoms from multiple sources such as those listed in DSM-5, the Bush Francis Catatonia Rating Scale, and those that have been reported in the literature, allowed us to achieve greater diagnostic consensus and address the heterogeneity in the presentation of catatonia (Schorr et al. 2024). Despite its limitations, this study identifies barriers to the diagnosis of catatonia in persons with ASD/NDD and proposes recommendations to increase diagnostic reliability, which could have positive implications for clinical practice, education, and research.

## References

[CR1] American Psychiatric Association. (2013). *Diagnostic and statistical manual of mental disorders* (5th ed.). Author.

[CR2] Anand, S., Kumar Paliwal, V., Singh, L. S., & Uniyal, R. (2019). Why do neurologists miss catatonia in neurology emergency? A case series and brief literature review. *Clinical Neurology and Neurosurgery*, *184*, 105375. 10.1016/j.clineuro.105375. PMID: 31147176. 10.1016/j.clineuro.2019.105375 31147176

[CR3] Breen, J., & Hare, D. J. (2017). The nature and prevalence of catatonic symptoms in young people with autism. *Journal of Intellectual Disability Research.* 61*(6)*:580–593. 10.1111/jir.12362. PMID: 28150394. 10.1111/jir.12362 28150394

[CR4] Bush, G., Fink, M., Petrides, G., Dowling, F., & Francis, A. (1996). Catatonia. I. Rating scale and standardized examination. *Acta Psychiatrica Scandinavica*, *93*(2), 129–136. 10.1111/j.1600-0447. PMID: 8686483. 8686483 10.1111/j.1600-0447.1996.tb09814.x

[CR5] Dhossche, D. M., Reti, I. M., Shettar, S. M., & Wachtel, L. E. (2010). *Journal of ECT*, *26*(4), 266–9. 10.1097/yct.0b013e3181cb5f60.PMID: 21155151. 10.1097/yct.0b013e3181cb5f60 21155151

[CR6] Fink, M. (2011). Catatonia from its creation to DSM-V: Considerations for ICD. *Indian Journal of Psychiatry*, *53*(3), 214–7. 10.4103/0019-5545.86810. PMID: 22135438. 10.4103/0019-5545.86810 PMC3221176 22135438

[CR7] Ghaziuddin, N., Dhossche, D., & Marcotte, K. (2012). Retrospective chart review of catatonia in child and adolescent psychiatric patients. *Acta Psychiatrica Scandinavica*, *125*(1), 33–38. 10.1111/j.1600-0447.2011.01778.xEpub 2011 Nov.1. PMID:22040029. 22040029 10.1111/j.1600-0447.2011.01778.x

[CR8] Hallyburton, A. (2022). Diagnostic overshadowing: An evolutionary concept analysis on the misattribution of physical symptoms to pre-existing psychological illnesses. *International Journal of Mental Health Nursing*, *31*(6), 1360–1372. 10.1111/inm.13034Epub 2022 PMID: 35718951; PMCID: PMC9796883. 35718951 10.1111/inm.13034 PMC9796883

[CR9] Ishizuka, K., Tachibana, M., & Inada, T. (2022). Possible commonalities of clinical manifestations between dystonia and catatonia. *Frontiers in Psychiatry Apr*, *29*, 13:876678. 10.3389/fpsyt.2022.876678PMID: 35573366; PMCID: PMC9098969. 10.3389/fpsyt.2022.876678 PMC9098969 35573366

[CR10] Llesuy, J. R., Medina, M., Jacobson, K. C., & Cooper, J. J. (2018). Catatonia under-diagnosis in the General Hospital. *The Journal of Neuropsychiatry and Clinical Neurosciences*, *30*(2), 145–151. 10.1176/appi.neuropsych.17060123. Epub 2018 Jan 12. PMID: 29325478. 10.1176/appi.neuropsych.17060123 29325478

[CR11] Northoff, G. (2002). What catatonia can tell Us about top-down modulation: A neuropsychiatric hypothesis. *Behavioral Brain Sciences*, *25*, 555–604. 12958742 10.1017/s0140525x02000109

[CR12] Oldham, M. (2019). A diversified theory of catatonia. *Lancet Psychiatry*, *6*(7), 554–555. 10.1016/S2215-0366(19)30212-3. Epub 2019 Jun 10. PMID: 31196792. 10.1016/S2215-0366(19)30212-3 31196792

[CR13] Partl, S., Pfuhlmann, B., Jabs, B., & Stober, G. (2011). Meine krankheit Ist eine Kopfkrankheit, die Schwer Zudefinieren Ist. *Der Nervenarzt*, *82*, 67–78. 21274695 10.1007/s00115-009-2914-y

[CR14] Rasmussen, S. A., Mazurek, M. F., & Rosebush, P. I. (2016). Catatonia: Our current Understanding of its diagnosis, treatment and pathophysiology. *World Journal of Psychiatry*, *6*(4), 391–398. 10.5498/wjp.v6.i4.391 28078203 10.5498/wjp.v6.i4.391 PMC5183991

[CR15] Reinfeld, S., & Gill, P. (2023). Diagnostic overshadowing clouding the efficient recognition of pediatric catatonia: A case series. *CNS Spectrum*, *28*(5), 587–591. Epub 2022 28. PMID: 36440510. 10.1017/S1092852922001158 36440510

[CR16] Schorr, B., Clauss, J. M. E., de Billy, C. C., Dassing, R., Zinetti-Bertschy, A., Domergny-Jeanjean, L. C., Obrecht, A., Mainberger, O., Schürhoff, F., Foucher, J. R., & Berna, F. (2024). Subtyping chronic catatonia: Clinical and neuropsychological characteristics of progressive periodic catatonia and chronic system catatonias vs. non-catatonic schizophrenia. *Schizophrenia Research*, *263*, 55–65. Epub 2022 Nov 18. PMID: 36411196. 36411196 10.1016/j.schres.2022.10.009

[CR17] Tuerlings, J. H., van Waarde, J. A., & Verwey, B. (2010). A retrospective study of 34 catatonic patients: Analysis of clinical care and treatment. *General Hospital Psychiatry*, *32*, 631–635. 21112456 10.1016/j.genhosppsych.2010.08.007

[CR18] Walther, S., & Strick, W. (2016). Catatonia. *CNS Spectrums*, *21*, 341–348. 27255726 10.1017/S1092852916000274

[CR19] Wing, L., & Shah, A. (2006). A systematic examination of catatonia-like clinical picture in autism spectrum disorders. *International Review of Neurobiology*, *72*, 21–39. 16697289 10.1016/S0074-7742(05)72002-X

